# Terpinen-4-ol as an Antibacterial and Antibiofilm Agent against *Staphylococcus aureus*

**DOI:** 10.3390/ijms21124531

**Published:** 2020-06-25

**Authors:** Laísa Cordeiro, Pedro Figueiredo, Helivaldo Souza, Aleson Sousa, Francisco Andrade-Júnior, Daianne Medeiros, Jefferson Nóbrega, Daniele Silva, Evandro Martins, José Barbosa-Filho, Edeltrudes Lima

**Affiliations:** 1 Department of Pharmaceutical Science, Federal University of Paraíba, João Pessoa 58033-455, Brazil; pedrotrfigueiredo@gmail.com (P.F.); aleson_155@hotmail.com (A.S.); juniorfarmacia.ufcg@outlook.com (F.A.-J.); daiannemedeiros1@gmail.com (D.M.); jeffersonrodriguesn@hotmail.com (J.N.); danielefigueredo31@gmail.com (D.S.); jbarbosa@ltf.ufpb.br (J.B.-F.); edelolima@yahoo.com.br (E.L.); 2 Chemistry Department, Federal University of Paraíba, João Pessoa 58033-455, Brazil; helivaldog3@gmail.com; 3 Chemistry Department, State University of Piauí, Piripiri 64260-000, Brazil; evandropaulo@prp.uespi.br

**Keywords:** terpinen-4-ol, *Staphylococcus aureus*, antibacterial, antibiofilm, molecular docking, checkerboard method

## Abstract

*Staphylococcus aureus* is able to rapidly develop mechanisms of resistance to various drugs and to form strong biofilms, which makes it necessary to develop new antibacterial drugs. The essential oil of *Melaleuca alternifolia* is used as an antibacterial, a property believed to be mainly due to the presence of terpinen-4-ol. Based on this, the objective of this study was to evaluate the antibacterial and antibiofilm potential of terpinen-4-ol against *S. aureus*. The Minimal Inhibitory and Minimal Bactericidal Concentrations (MIC and MBC) of terpinen-4-ol were determined, and the effect of its combination with antibacterial drugs as well as its activity against *S. aureus* biofilms were evaluated. In addition, an in silico analysis of its pharmacokinetic parameters and a molecular docking analysis were performed. Terpinen-4-ol presented a MIC of 0.25% (*v/v*) and an MBC of 0.5% (*v/v*) (bactericidal action); its association with antibacterials was also effective. Terpinen-4-ol has good antibiofilm activity, and the in silico results indicated adequate absorption and distribution of the molecule in vivo. Molecular docking indicated that penicillin-binding protein 2a is a possible target of terpinen-4-ol in *S. aureus*. This work highlights the good potential of terpinen-4-ol as an antibacterial product and provides support for future pharmacological studies of this molecule, aiming at its therapeutic application.

## 1. Introduction

*Staphylococcus aureus* is a Gram-positive bacterial species capable of efficiently circumventing the body’s defense barriers, through its virulence mechanisms [1]. This pathogen can cause a wide range of infections and has a high rate of morbidity and mortality, which generates high costs for health systems [2]. Although *S. aureus* is naturally susceptible to practically all antibiotics that have already been developed, it is also capable of rapidly developing mechanisms of resistance to various drugs, compromising the use of even the most potent antibacterials agents, such as methicillin and vancomycin [3]. Despite the spreading of bacterial drug resistance, few antibacterial drugs have been introduced in recent years. This means that there is a high need for new drugs to treat infections [4].

Another complicating factor in infections caused by *S. aureus* is the strong ability of this pathogen to form biofilms, which occur in sites that are difficult to access and are highly resistant to drugs, contributing to persistent infections [5]. In addition to the fact that few drugs are effective against biofilms, some drugs may even stimulate the formation of this virulence factor when they are in subinhibitory concentrations, making the treatment of infections even more difficult [6]. 

In view of the constant evolution of drug-resistant microorganisms and the limited existence of products with antibiofilm activity, it is necessary to develop new therapeutic alternatives. In this context, medicinal plants are one of the main sources of bioactive molecules for antimicrobial purposes. Natural products can show considerable efficacy against biofilms formed by different types of bacteria [7]. Such molecules usually have reduced toxic effects and are capable of acting against strains resistant to conventional drugs, in addition to serving as a prototype for various molecular modifications aimed at improving their activity [8]. 

Among the plant species with remarkable antimicrobial activity *Melaleuca alternifolia* (Maiden & Betche) Cheel is used in traditional medicine in various parts of the world, including Asia, America, and Australia. Its essential oil is used to prepare soaps, shampoos, and other cosmetic products to fight superficial infections. The essential oil of *M. alternifolia* is known as “tea tree oil” and contains more than 100 components, such as monoterpenes, sesquiterpenes, and phenolic compounds. The most abundant substance in tea tree essential oil is terpinen-4-ol, which is present in an amount of at least 30% [9].

The activity of terpinen-4-ol has been described in the literature, and it is believed that the substance is primarily responsible for the antibacterial activity of the essential oil of *M. alternifolia*, as there is a similarity in the antibacterial efficacy of the essential oil and the isolated compound [9,10,11,12]. In addition to the antibacterial activity of terpinen-4-ol, good antibiofilm activity of the substance was observed against some biofilm-forming species, which highlights its potential for application in clinical practice [10,13,14]. However, there are still few studies on the activity of terpinen-4-ol against biofilms formed by *S. aureus*.

Based on this, the aim of this study was to evaluate the antibacterial activity of terpinen-4-ol against *S. aureus,* investigating the possible molecular target of the substance through molecular docking analysis. We also aimed to evaluate the effect of its combination with antibacterials commonly used in clinical practice and the antibiofilm potential of terpinen-4-ol against *S. aureus*, in addition to performing a preliminary in silico analysis of its pharmacokinetic parameters. Thus, this study is expected to contribute to elucidate the biological activities of terpinen-4-ol, encouraging the investigation of this substance as an alternative treatment for infections caused by *S. aureus*, as well as a prototype for structural modifications that enhance its biological activity.

## 2. Results and Discussion

### 2.1. Determination of Minimum Inhibitory Concentration (MIC) and Minimum Bactericidal Concentration (MBC) of Terpinen-4-ol Against S. aureus

The antibacterial activity of terpinen-4-ol on *S. aureus* was assessed by determining the Minimum Inhibitory Concentration (MIC) and Minimum Bactericidal Concentration (MBC); the results are shown in Table 1.

The MIC of terpinen-4-ol was 0.25 % (*v/v*) against all tested strains (Table 1). When analyzing the MBC, the value obtained was 0.5 % for all strains, which corresponds to a MIC/MBC ratio of 1:2. A bactericidal substance is capable of killing bacterial cells, while bacteriostatic substances inhibit or slow their growth without causing death. A drug is considered to exhibit bactericidal activity when the MIC/MBC ratio is ≤4 [15,16]. Thus, terpinen-4-ol has bactericidal properties against the strains of *S. aureus* analyzed in this study.

These results corroborate the data of Loughlin et al. [17], who used terpinen-4-ol against methicillin-resistant *S. aureus* (MRSA) and identified a MIC of 0.25% (*v/v*) and an MBC of 0.5% (*v/v*) for 90% of the strains analyzed, showing the bactericidal potential of this substance. Hammer et al. [18] also identified a MIC of 0.25% against reference strains and *S. aureus* clinical isolates. However, studies using isolated terpinen-4-ol against *S. aureus* are still few in the scientific literature.

Although data on the bacteriostatic or bactericidal activity of a substance can provide valuable information on the potential action of antibacterial agents in vitro, these results are applicable to experiments in standardized in vitro conditions and may vary according to the type of bacteria, quantity of inoculum, and duration of the tests. These variations are, therefore, observed in clinical practice, where the conditions encountered are as varied as possible [16]. Thus, we decided to combine the information obtained from in vitro experiments with pharmacokinetic and pharmacodynamic data to provide a more significant prediction of the in vivo efficacy of terpinen-4-ol.

### 2.2. Molecular Docking Analysis

A previous in silico screening was performed through molecular docking, in order to identify the molecular targets of terpinen-4-ol in *S. aureus*. However, the analyses did not show significant interactions that could clarify the way in which terpinen-4-ol exerts the antibacterial activity observed in this study. Since the in silico analyses of terpinen-4-ol on *S. aureus* did not yield clear results, in vitro and in vivo studies must be performed to fully elucidate the mechanism of action of this substance in *S. aureus*.

On the other hand, molecular docking showed a possible interaction between terpinen-4-ol and penicillin-binding protein 2a (PBP2a), which is one of the main molecules involved in *S. aureus* resistance to beta-lactam drugs due to its low affinity for them and to its transpeptidase activity that allows cell wall biosynthesis [19,20].

**Figure 1:**
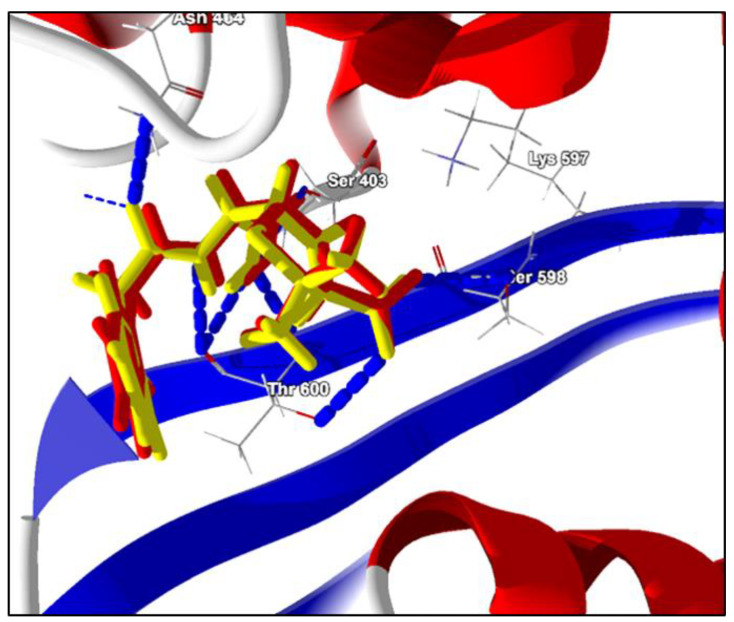
Overlapping a Protein Data Bank (PDB) ligand (Open Form, Penicillin G) with the best redocking conformation. Red: PDB binder. Yellow: conformation of the ligand after redocking. Blue-dotted: hydrogen bonding interactions.

For the validation of the protocols performed in molecular docking, redocking was performed, and the value of root-mean standard deviation (RMSD) was used to analyze the accuracy of the results. To be considered a valid docking, the RMSD values must remain in the range of 0–2 Å [21]. As noted, the RMSD value was 0.38 Å for the tested enzyme, thus within acceptable values. This result was confirmed by overlapping the co-crystallized ligands with the best conformation after re-docking (Figure 1).

**Figure 2:**
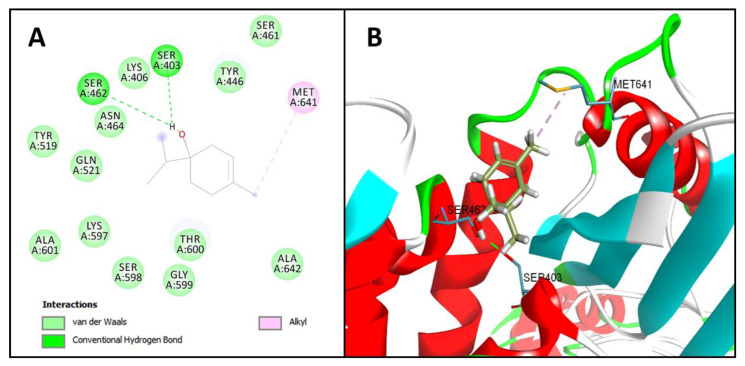
Two-dimensional (**A**) and three-dimensional; (**B**) representation of interactions between terpinen-4-ol and penicillin-binding protein 2a (PBP2a) active site.

When analyzing the molecular docking results, it appeared that the hydroxyl of terpinen-4-ol makes two hydrogen bonds with the amino acid residues Ser403 and Ser462. The terpinen-4-ol also established 12 Van Der Walls interactions and one alkyl interaction with Met641 (Figure 2). All these interactions are necessary for terpinen-4-ol to efficiently anchor to the active site of the PBP2a enzyme (Table 2).

It is relevant to note that the interactions of terpinen-4-ol with Lys406 and Thr600 residues are especially important (Figure 2), as they are essential for the proper anchoring of the molecule in the active site of the enzyme and corresponds to two of the three main bonds (Ser403, Lys 406 and Thr 600) made by penicillin G (Protein Data Bank (PDB) ligand) with the enzyme’s active site thus inhibiting PBP2a, as demonstrated by Lim and Strynadka (2002) [22].

It was found that *S. aureus* resists beta-lactams by two main mechanisms, i.e., by using beta-lactamases to inactivate the antibiotic and, more critically, through the use of a low-affinity PBP2a. The production of PBP2a is the main mechanism developed by MRSA inducing broad clinical resistance to beta-lactams. MRSA resistance is mediated through the acquisition of a gene cassette containing the gene *mec*A, which encodes the low-affinity altered transpeptidase PBP2a [19,20].

Thus, the binding of terpinen-4-ol to PBP2a and the consequent inhibition of its activity can be an effective adjuvant tool in the treatment of resistant strains using beta-lactams. Future studies should be carried out to better explore this mechanism, investigating this possible action in vitro and in vivo, using *S. aureus* resistant strains. 

### 2.3. Association Test

As can be seen in Table 3, the effects of the association of terpinen-4-ol with the antibacterial drugs gentamicin, cefazolin, vancomycin, oxacillin, and meropenem varied according to the type of combination. Terpinen-4-ol showed synergism when associated with cefazolin, oxacillin, and meropenem. On the other hand, the effect was unchanged when terpinen-4-ol was combined with gentamicin and vancomycin (Table 3).

The combination of natural and antimicrobial products has proven to be quite effective and promising in the clinic. Combining substances makes it possible to reduce the required administration dose of each medication and, therefore, to reduce dose-dependent toxic effects. Another benefit of combining natural products with antimicrobial drugs is the possibility of effectively fighting resistant strains. Phytoconstituents can act as bacterial resistance-modifying agents, restoring the effectiveness of commercial drugs. This may be due to the different mechanisms of action of natural products, such as inhibition of target-modifying and drug-degrading enzymes or of efflux pumps [23,24,25].

Terpinen-4-ol demonstrated an excellent combinatory effect with cefazolin, oxacillin, and meropenem, which suggests the possibility of reducing the viability of the bacterial strains using lower concentrations of both substances in combination. It is also possible, therefore, to reduce the side effects resulting from the administration of these drugs. The lack of effect found for the association with gentamicin and vancomycin is also relevant, as it demonstrates that terpinen-4-ol does not interfere negatively with these drugs during treatment.

Considering the results of molecular docking, indicating inhibition of PBP2a, and the promising results obtained using drug combinations, future studies should examine the effects of the association of terpien-4-ol with other drugs on drug-resistant strains, to possibly identify drug combinations capable of altering the resistance of these bacteria.

### 2.4. Antibiofilm Effect

**Figure 3:**
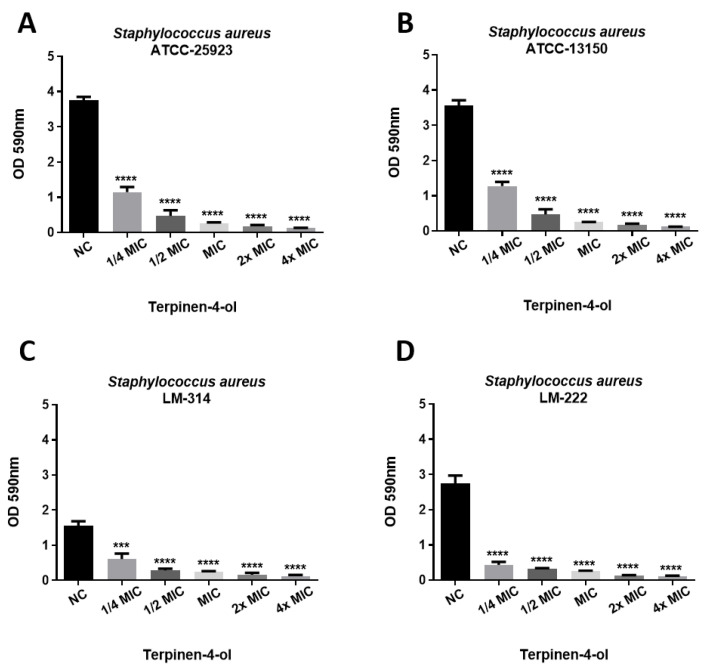
Effect of terpinen-4-ol on the inhibition of biofilm formation by different *S. aureus* strains: (**A**) ATCC-25923; (**B**) ATCC-13150; (**C**) LM-314; (**D**) LM-222. NC: negative control. Statistical analysis, compared to the negative control: **** *p* ≤ 0.0001; *** *p* ≤ 0.001. MIC: Minimum Inhibitory Concentration.

The antibiofilm activity of terpinen-4-ol was also assessed. As seen in Figure 3, terpinen-4-ol showed a strong ability to inhibit the formation of biofilm by *S. aureus*, at all tested concentrations. Significant differences were observed between the treated groups and the control group without treatment, indicating that terpinen-4-ol was able to act on the preformed biofilm even at subinhibitory concentrations in a dose-dependent manner (Figure 3). The percentage of biofilm formation inhibition was greater than 80% at 0.5MIC and approximately 90% at concentrations corresponding to 2MIC and 4MIC.

The strong antibiofilm activity of terpinen-4-ol against *S. aureus* found in this study corroborates data by other authors showing the activity of this phytoconstituent against biofilms formed by different species. Studies have demonstrated the excellent potential of terpinen-4-ol against biofilms formed by oral pathogens such as *Porphyromonas gingivalis, Fusobacterium nucleatum* [26], *Streptococcus mutans*, and *Lactobacillus acidophilus* [10]. In addition, it was observed that this activity extends against other pathogens [27], including *Pseudomonas aeruginosa* [28]. However, the effect of terpinen-4-ol on biofilms formed by *S. aureus* is still scarcely explored. 

It is important to note that terpinen-4-ol was able to inhibit biofilm formation in a concentration-dependent manner, even at sub-MIC concentrations. Assessing the antibiofilm effect at subinhibitory concentrations is a clinically relevant process, because, during infection treatment, a part of microorganism populations is exposed to suboptimal concentrations, even when the recommended conditions of use of the drugs are followed [6,29].

Some classes of antibiotics used in clinical practice, including β-lactams, are capable of promoting the formation of biofilms at subinhibitory concentrations, as they lead to changes in the expression of bacterial virulence factors. Thus, low doses of these drugs can interfere with the course of an infection, making it difficult to treat these diseases [6,29,30,31,32]. This was not observed with terpinen-4-ol against *S. aureus*, which emphasizes a good clinical potential of this substance against biofilms. 

There is a great interest in to preventing the formation of biofilm by pathogenic species. However, few studies have been carried out to assess the potential for rupture of already formed biofilms which, once established, are difficult to remove and are responsible for high rates of therapeutic failure [33].

**Figure 4:**
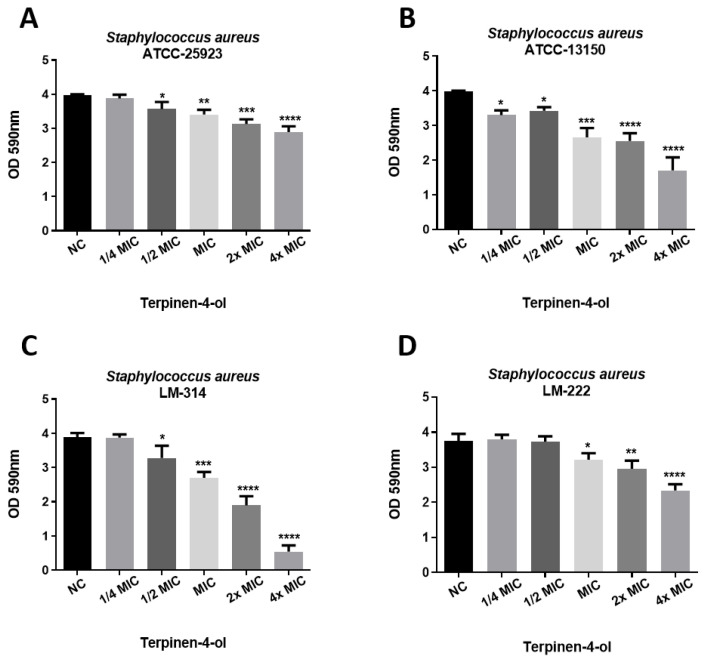
Effect of terpinen-4-ol on preformed biofilms by different *S. aureus* strains: (**A**) ATCC-25923; (**B**) ATCC-13150; (**C**) LM-314; (**D**) LM-222. NC: negative control. Statistical analysis compared to the negative control: **** *p* ≤ 0.0001; *** *p* ≤ 0.001; ** *p* ≤ 0.01, * *p* ≤ 0.05.

On the basis of terpinen-4-ol showed good activity in inhibiting the formation of biofilm by *S. aureus*, its potential for breaking previously formed biofilms was also investigated (Figure 4). Although it was possible to observe a significant difference between the treated groups in relation to the negative control, the activity of terpinen-4-ol on preformed biofilms was lower than its ability to inhibit the formation of biofilms, as shown in Figure 3.

This was expected, due to the fact that biofilms are already mature and, therefore, more difficult to eliminate. Thus, terpinen-4-ol is more effective in inhibiting the formation of *S. aureus* biofilms than in breaking/eliminating already formed biofilms. 

The ability of some microorganisms, such as *S. aureus*, to form biofilms contributes to their antibacterial resistance and to therapeutic failures [34]. The development of effective tools to remove biofilms not only improves the treatment of biofilm-related infections but can also potentially offer benefits to slow the spread of antibiotic resistance [35].

The results obtained in this study show that terpinen-4-ol is a potential antibiofilm agent, being able to prevent biofilm formation, even at suboptimal concentrations, in addition to acting on already established biofilms. Thus, it is relevant that studies on this activity of terpinen-4-ol be further developed, evaluating its activity against biofilms formed by other bacterial species and elucidating its mechanism of action.

### 2.5. In Silico Analysis of Pharmacokinetic Parameters

In this work, an in silico study was carried out in order to verify the theoretical absorption of terpine-4-ol. According to the values of the physical-chemical parameters presented in Table 4, calculated using the online program SwissADME, it was possible to predict whether the terpine-4-ol molecule could be developed as a drug, based on the rules of Lipinski [36], Ghose [37], Veber [38], and Egan [39]. The data obtained are shown in Table 4.

The molecular mass of a substance is an important parameter, because the greater the molecular mass, the greater the volume of the substance, and the more difficult the passage of the molecule to the intracellular environment. The molecular mass of terpinen-4-ol is 154.25 g/mol and is in accordance with Lipinski’s rule. However, for Ghose [37], this value is well below the optimal range, which is 180 ≤ MW ≤ 480, so the molecule violates this parameter. Another important parameter is Log P, related to the hydrophobicity of a given compound and the ability to cross plasma membranes. The Log P obtained for the substance was 2.60 and met the rules according to Lipinski (Log Po/w ≤ 5), Ghose [37] (Log Po/w ≤ 5.6) and Egan [39] (Log Po/w ≤ 5.8). The results presented in Table 4 for the number of hydrogen acceptors and donors correspond to the Lipinski’s rule, with values for acceptors of 1 and for donors of 1. According to these values presented and compared according to the Lipinski’s rule of five, terpine-4-ol has an excellent theoretical oral bioavailability. However, Veber presents two essential parameters for molecules to be administered orally, which are Topological Polar Surface Area (TPSA) ≤ 140 Å and the number of rotatable connections. The molecule under study has a TPSA of 20.23 Å, and the number of rotatable connections is 1; therefore, the molecule has a high prospect of being used orally. Water solubility is an important feature for absorption and distribution of a drug in the body. The Log S (determined by the Ali method [40]) of terpine-4-ol is −2.78, indicating that the compound is soluble according to the classes shown in Table 4.

Inadequate penetration of the infection site is one of the main factors related to the failure of antibacterial therapies. The active drug needs to reach the bacteria in appropriate body fluids and tissues at concentrations necessary to kill or suppress pathogens’ growth. Even for substances with good in vitro activity, the pharmacokinetic parameters are decisive for its clinical use [16]. Thus, the results of this preliminary in silico analysis support the potential of terpinen-4-ol as a drug candidate, because in addition to displaying a strong antibacterial and antibiofilm activity, it presents pharmacokinetic parameters that suggest good absorption and biodistribution in vivo. Although this is a preliminary study, these results serve as a basis for future studies seeking to evaluate the absorption and biodistribution of this molecule in the human organism.

Based on all the results obtained, this study showed that terpinen-4-ol has strong antibacterial activity against *S. aureus*, acting in a bactericidal manner. This phytoconstituent probably acts by interfering with the synthesis of the bacterial cell wall, PBP2a being one of its targets. Its combination with antibacterials demonstrate promising effects for use in clinical practice. Terpinen-4-ol has a remarkable antibiofilm activity and is able to inhibit biofilm formation even at subinhibitory concentrations. The in silico results showed a pharmacokinetic profile indicating good theoretical oral bioavailability, good solubility, and adequate absorption and distribution of the molecule in vivo. Terpinen-4-ol has characteristics that suggest it is a good candidate as a drug to treat infections by *S. aureus*, whether isolated or associated with other drugs; it is also able to interfere with *S. aureus*-associated biofilms. This work encourages future pharmacological studies of this terpinen-4-ol, aiming at its therapeutic application.

## 3. Materials and Methods

### 3.1. Substances

Terpinen-4-ol and antibacterials (gentamicin, cefazolin, vancomycin, oxacillin, and meropenem) were obtained from Merck/Sigma-Aldrich^®^ (Darmstadt, Germany). These substances were solubilized in dimethyl sulfoxide (DMSO) at 5% and Tween-80 at 2%, to obtain emulsions in the concentrations necessary for the tests.

### 3.2. Strains

The clinical isolates strains of *S. aureus* used in this study belong to the MICOTECA of the Antibacterial and Antifungal Activity Research Laboratory of the Federal University of Paraíba, Brazil, which are: LM-02, LM-40, LM-222 and LM-232 (isolated from nasal discharges) and LM-45, LM-116, LM-297, and LM-314 (isolated from oropharyngeal secretions). The American Type Culture Collection strains ATCC-13150 and ATCC-25923 were used as controls. For use in the assays, bacterial suspensions were prepared in 0.9% saline solution from fresh cultures and adjusted to the McFarland standard 0.5 scale.

### 3.3. Minimum Inhibitory Concentration (MIC)

MIC determination was performed using the broth microdilution technique (cation-adjusted Mueller–Hinton broth) in a 96-well plate to obtain different concentrations of terpinen-4-ol [41]. In parallel, controls for sterility, cell viability, and interference of the vehicles used in the preparation of terpinen-4-ol emulsions (DMSO and Tween-80) were also performed. MIC is defined as the lowest concentration capable of causing complete inhibition of bacterial growth after 24 h at 35 ± 2 °C.

### 3.4. Minimum Bactericidal Concentration (MBC)

The MBC was determined after the MIC reading, removing aliquots from wells where there was no visible growth (supra-inhibitory concentrations) and inoculating them in new plates containing only culture broth [15,42]. All controls were performed. MBC is defined as the lowest concentration capable of causing complete inhibition of bacterial growth after 24 h at 35 ± 2 °C.

### 3.5. Molecular Docking

The geometry of the chemical structure of terpinen-4ol was optimized using the Hyperchem program (v. 8.0.6, Hypercube^®^, Gainesville, FL, USA), using the molecular mechanics method (MM+) and the semi-empirical method AM1 (Austin Model 1) [43]. Molecular docking was used to verify if terpinen-4-ol can interact with several molecular targets of *S. aureus*. Previous in silico screening was carried out using 11 enzymes that are targets of different classes of antimicrobials. All enzyme structures were obtained from Protein Data Bank (https://www.rcsb.org/). In silico screening showed a preferred interaction of terpinen-4-ol with the enzyme PBP2a, and for this reason, PBP2a is the enzyme explored in this article.

PBP2a enzyme structure was obtained from Protein Data Bank (PDB) (https://www.rcsb.org/) together with its co-crystallized inhibitor (open form, Penicillin G), with ID PDB: 1MWT (2.2 Å) [21]. Molecular docking was performed using the Molegro Virtual Docker (MVD) software (v. 6.0.1, Molegro ApS^®^, Aarhus, Denmark) using the following parameters: scoring function: MolDock Score; ligand evaluation: internal ES, H-bond internal, Sp2-Sp2 torsions, number of executions: 10; algorithm: MolDock SE; maximum interactions: 1500; maximum population size: 50; maximum steps: 300; neighborhood factor: 1.00; maximum number of conformations: 5. Water molecules were removed, and a template was generated for the PDB enzyme-co-crystallized inhibitor.

### 3.6. Association Test

The association test by the checkerboard method was performed using terpinen-4-ol in combination with gentamicin, cefazolin, vancomycin, oxacillin, and meropenem against *S. aureus* (ATCC-25923, ATCC-13150, LM-222 and LM-314). Different concentrations of terpinen-4-ol (8 × MIC, 4 × MIC, 2 × MIC, MIC, 0.5 × MIC, 0.25 × MIC and 0.125 × MIC) were combined with different concentrations of antibacterials (8 × MIC, 4 × MIC, 2 × MIC, MIC, 0.5 × MIC, 0.25 × MIC and 0.125 × MIC) and the bacterial inoculum was added. All controls were performed in parallel. The reading of the experiment was performed after incubation at 35 ± 2 °C for 24 h to observe the presence or absence of visible bacterial growth. The effect produced by the combination of substances was evaluated by determining the fractional inhibitory concentration index (FICI). This index was calculated summing up thr fractional inhibitory concentrations (FIC), where FIC_A_ = (MIC of substance A in combination)/(MIC of substance A alone), and FIC_B_ = (MIC of substance B in combination)/(MIC of substance B alone); thus FICI = FIC_A_ + FIC_B_. The association was defined as synergistic for FICI ≤ 0.5, as additive for 0.5 < FICI < 1, as indifferent (unchanged) for 1 ≤ FICI < 4, and as antagonistic for FICI ≥ 4 [42,44].

### 3.7. Antibiofilm Effect

The evaluation of the potential of terpinen-4-ol to inhibit the formation of *S. aureus* (ATCC-25923, ATCC-13150, LM-222 and LM-314) biofilm was analyzed, as well as its ability to act against preformed biofilms. To assess the potential for inhibition of biofilm formation, microdilution plates were incubated statically with cerebral heart infusion broth (BHI) containing different concentrations of terpinen-4-ol in the presence of the bacterial inoculum (final concentration 10^7^ colony-forming units (CFU/mL)). The negative control contained only culture broth and inoculum. After 24 h of incubation at 35 ± 2 °C, the contents of the wells were discarded, and the plates were washed gently with sterile distilled water, in order to remove planktonic cells, and then dried at room temperature. After drying, a 1% crystal violet solution was added and left for 40 min. The dye was discarded, and its excess on the wells’ walls was removed by washing with distilled water. Once dry, absolute ethanol was added to the wells and, after 30 min, the plates were read on a microplate spectrophotometer (Multiskan GO, Thermo Fisher Scientific Corporation^®^, Vantaa, Finland) at 590 nm [45]. All analyses were performed in quintuplicate. Statistical significance was determined by pair-wise testing using Student’s *t*-test, and statistical significance was accepted for *p* values < 0.05. The percentage of biofilm inhibition was calculated using the following Equation (1):

(1) % biofilm inhibition=[(OD590 control−OD590 test)/OD590 control]×100

To analyze the potential of terpinen-4-ol to act against preformed biofilms, the plates were previously incubated with BHI broth and inoculum (final concentration 10^7^ CFU/mL) for 24 h at 35 ± 2 °C, in order for the biofilms to form in the wells. Then, the contents of the wells were removed, and fresh culture broth containing different concentrations of terpinen-4-ol was added. Again, the plates were incubated statically under the same conditions of time and temperature. Then, the same process described above was performed before evaluation with a microplate spectrophotometer [46]. Statistical significance was determined by pair-wise testing using the Student’s t-test, and the results were considered statistically significant when *p* < 0.05 for the rejection of the null hypothesis. For this plotting, GraphPad Prism (v. 6.0 for Windows, GraphPad Software^®^, San Diego, CA, USA) software was used.

### 3.8. In Silico Analysis of Pharmacokinetic Parameters

Violations of the rules of Lipinski [36], Ghose [37], Veber [38], and Egan [39] help to evaluate the pharmacokinetic characteristics of drug candidates. The following parameters were evaluated for terpinen-4-ol: physicochemical properties, lipophilicity, water solubility, and druglikeness, using the free online software SwissADME (www.swissadme.ch) (Swiss Institute of Bioinformatics^®^, Lausanne, Switzerland). Such results were analyzed using the rules of Lipinski [36], Ghose [37], Veber [38], and Egan [39].

## Figures and Tables

**Table 1:** Minimal Inhibitory Concentration (MIC), Minimal Bactericidal Concentration (MBC), and classification of the effect of terpinen-4-ol against *Staphylococcus aureus* strains.

*S. aureus*	Terpinen-4-ol			
	MIC	MBC	MIC/MBC	Effect
ATCC-25923	0.25% (*v/v*)	0.5% (*v/v*)	1:2	Bactericidal
ATCC-13150	0.25% (*v/v*)	0.5% (*v/v*)	1:2	Bactericidal
LM-02	0.25% (*v/v*)	0.5% (*v/v*)	1:2	Bactericidal
LM-40	0.25% (*v/v*)	0.5% (*v/v*)	1:2	Bactericidal
LM-45	0.25% (*v/v*)	0.5% (*v/v*)	1:2	Bactericidal
LM-116	0.25% (*v/v*)	0.5% (*v/v*)	1:2	Bactericidal
LM-222	0.25% (*v/v*)	0.5% (*v/v*)	1:2	Bactericidal
LM-232	0.25% (*v/v*)	0.5% (*v/v*)	1:2	Bactericidal
LM-297	0.25% (*v/v*)	0.5% (*v/v*)	1:2	Bactericidal
LM-314	0.25% (*v/v*)	0.5% (*v/v*)	1:2	Bactericidal

**Table 2:** Free binding energies and targeting residues of the PDB ligand and terpinen-4-ol in the interaction with PBP2a.

	Moldock Score	Targeting Residues
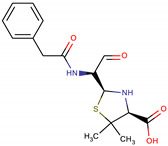 Open Form, Penicillin G (PDB ligand)	−125.7 kcal/mol	Hydrogen bonds: Ser403, Thr600, Lys597, Ser598, Asn464 e Ser462 Hydrophobic interactions: Tyr446 Van Der Walls: Lys406, Lys597, Gly599, Ala601, Ala642 e Met641.
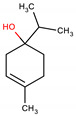 Terpinen-4-ol	−54.8 kcal/mol	Hydrogen bond: Ser403 e Ser462. Hydrophobic interactions: Met641. Van Der Walls: Lys406, Asn464, Tyr446, Gln521, Thr600, Ser461, Lys406, Tyr519, Lys597, Ala601, Ser598, Gly599, Ala642

**Table 3:** Terpinen-4-ol in association with different antibacterial drugs against *S. aureus.*

Strains and Drugs	FICI	Effect ^1^
**ATCC-25923**		
Gentamicin	1.06	Indifference
Cefazolin	0.50	Synergism
Vancomycin	1.75	Indifference
Oxacillin	0.32	Synergism
Meropenem	0.50	Synergism
**ATCC-13150**		
Gentamicin	1.12	Indifference
Cefazolin	0.50	Synergism
Vancomycin	1.25	Indifference
Oxacillin	0.32	Synergism
Meropenem	0.50	Synergism
**LM-222**		
Gentamicin	1.06	Indifference
Cefazolin	0.32	Synergism
Vancomycin	1.25	Indifference
Oxacillin	0.32	Synergism
Meropenem	0.50	Synergism
**LM-314**		
Gentamicin	1.00	Indifference
Cefazolin	0.32	Synergism
Vancomycin	1.25	Indifference
Oxacillin	0.32	Synergism
Meropenem	0.50	Synergism

^1^ Synergism: Fractional Inhibitory Concentration Index (FICI) ≤ 0.5, additivity: 0.5 < FICI < 1, indifference: 1 ≤ FICI < 4, and antagonism: FICI ≥ 4.

**Table 4:** In silico studies of Lipinski’s parameters of terpinen-4-ol.

Parameters	Terpinen-4-ol
**Physicochemical Properties**	
Formula	C_10_H_18_O
Molecular Weight	154.25 g/mol
Num. Heavy atoms	11
Fraction Csp3	0.80
Num. Rotatable Bonds	1
Num. H-bonds acceptors	1
Num. H-bonds donors	1
Molar Refractivity	48.80
TPSA ^1^	20.23 Å ^2^
**Lipophilicity**	
Consensus ^2^ Log P_o/w_^3^	2.60
**Water Solubility**	
Log S (Ali)	−2.78
Class ^4^	Soluble
**Druglikeness**	
Lipinski ^5^	Yes; 0 violation
Ghose ^6^	No; 1 violation: MW < 160
Veber ^7^	Yes; 0 violation
Egan ^8^	Yes; 0 violation
Bioavailability Score	0.55

^1^ TPSA: Topological Polar Surface Area; ^2^ Consensus Log P_o/w_ = Average of all five predictions; ^3^ Log P_o/w_ = partition coefficient between n-octanol/water; ^4^ Class = Ali classes: insoluble < −10 < poor < −6 < moderately soluble < −4 < soluble < −2 < very soluble < 0 < highly; ^5^ Lipinski = MM ≤ 500; Log P_o/w_ ≤ 5; H-bond donors ≤ 5; H-bond acceptors ≤ 10;.^6^ Ghose = 180 ≤ MM ≤ 480; 20 ≤ No. of atoms ≤ 70; 40 ≤ Molar Refractivity ≤ 130; −0.4 ≤ Log P_o/w_ ≤ 5.6; ^7^ Veber = Num. Rotatable Bonds ≤ 10; TPSA ≤ 140 Å^2^; ^8^ Egan = Log P_o/w_ ≤ 5.88; TPSA ≤ 131.6 Å^2^.

## Abbreviations

ATCC	American Type Culture Collection
BHI	Cerebral Heart Infusion (Broth)
CFU	Colony-Forming Units
DMSO	Dimethyl Sulfoxide
FIC	Fractional Inhibitory Concentration
FICI	Fractional Inhibitory Concentration Index
MBC	Minimum Bactericidal Concentration
MIC	Minimum Inhibitory Concentration
MVD	Molegro Virtual Docker
MW	Molecular Weight
PDB	Protein Data Bank
RMSD	Root-Mean Standard Deviation
TPSA	Topological polar surface area
